# Patient Perspectives on the Course of Alcohol Use After Metabolic and Bariatric Surgery: Implications for Prevention, Intervention, and Future Research

**DOI:** 10.1111/cob.70061

**Published:** 2025-12-18

**Authors:** Erin N. Haley, Alyssa M. Vanderziel, Jordan M. Braciszewski, Heidi Westerman, Roland S. Moore, Kristina M. Jackson, Aaron Hamann, Arthur M. Carlin, Lisa R. Miller‐Matero

**Affiliations:** ^1^ College of Human Medicine Michigan State University East Lansing Michigan USA; ^2^ Center for Health Policy and Health Services Research Henry Ford Health System Detroit Michigan USA; ^3^ Behavioral Health Henry Ford Health System Detroit Michigan USA; ^4^ Pacific Institute for Research and Evaluation Berkeley California USA; ^5^ Department of Psychiatry Rutgers Robert Wood Johnson Medical School New Brunswick New Jersey USA; ^6^ Department of Surgery Henry Ford Health Detroit Michigan USA

**Keywords:** alcohol misuse, alcohol use disorder, metabolic and bariatric surgery, prevention and intervention, weight loss surgery

## Abstract

Despite substantial benefits of metabolic and bariatric surgery (MBS), up to 1 in 5 patients develop an alcohol use disorder (AUD) within 5 years post‐operatively. Cognitive‐behavioural processes that are relevant to the reinitiation and escalation of alcohol use post‐MBS are not well understood. Through content analysis of 20 patient interviews, we explored the course of alcohol use among individuals 6 months to 5 years post‐MBS and examined potential differences by level of use. Over half (55%) of the participants reported high alcohol use, most described a gradual reintroduction to alcohol post‐MBS, and escalation to misuse was thought to occur at an accelerated pace (i.e., potentially within months). Pre‐operative intentions, attitudes/beliefs about post‐MBS alcohol use, social factors, mood, visual cues, and post‐MBS biological changes were reported to influence the course of post‐MBS alcohol use. Although most features did not differ widely between those with higher versus lower alcohol consumption, reinitiating alcohol less than 12 months post‐operatively and certain attitudinal factors (i.e., minimising the risks) may relate to greater alcohol use post‐MBS. Findings illustrate the importance of early and long‐term monitoring of post‐MBS alcohol use. Several implications for early intervention and future research are discussed.

## Summary

- What Is Already Known About the Subject
  ○ The risk of alcohol misuse among those who have underwent MBS is high relative to the general population.
  ○ Several pre‐operative and post‐operative biopsychosocial risk factors for alcohol misuse post‐MBS have been identified.
  ○ There is an increase in the incidence of AUD 2 years following MBS, and this risk continues to rise over several years after MBS.
- What This Study Adds
  ○ Factors pertinent to post‐MBS alcohol use reinitiation, episodic use, and escalation to misuse among individuals up to 5‐years post‐MBS were explored.
  ○ Relevant intervention targets may include beliefs and attitudes about the risks of post‐MBS alcohol use, self‐monitoring skills, and alternative coping strategies.
  ○ Those with a gradual reintroduction to alcohol post‐MBS appeared to still be vulnerable to high levels of post‐MBS alcohol use.

## 1 Introduction

Metabolic and bariatric surgery (MBS) is the most effective treatment for severe obesity and associated comorbidities (i.e., diabetes) [1]. Despite considerable benefits, up to 20% of patients develop an alcohol use disorder (AUD) within the first 5 years post‐operatively [2, 3, 4]. Alcohol misuse may substantially undermine the benefits of MBS by contributing to recurrent weight gain and increasing the risk for major health consequences (e.g., nutritional deficiencies, cancer, pancreatitis, alcohol‐associated liver disease and cirrhosis, accidents/injuries, and psychiatric symptoms) [4, 5, 6]. While reducing the risk of AUD is crucial for this population, effective prevention and intervention options are limited. Pre‐surgical education on the risks of post‐MBS alcohol use alone is insufficient, and MBS surgery centre protocols for monitoring and supporting patient outcomes over the long term post‐MBS are variable [7, 8, 9, 10].

Increased risk of post‐MBS alcohol misuse has been explained by a combination of pre‐existing risk factors and complex biopsychosocial changes post‐operatively [3, 4, 6, 11]. For example, depression, low social support, pre‐operative alcohol misuse and maladaptive eating behaviours prior to MBS have been associated with post‐operative alcohol misuse [3, 12, 13]. Further, individuals undergo various biopsychosocial changes following MBS [3, 4, 14]. Biologically, changes in alcohol metabolism may increase sensitivity to alcohol and strengthen its reinforcing effects [4, 6]. Psychosocially, individuals can experience significant changes—which may be positive or challenging [14, 15]. For example, complex changes to one's self‐image (e.g., drastic weight loss, but the potential for excess skin), relationships (e.g., increased confidence and social opportunities, while some may be vulnerable to divorce) [16, 17], and lifestyle (e.g., required dietary changes to minimise physical discomfort and to maintain the benefits of MBS) may occur [14, 15, 18]. Relatedly, many patients have identified an unmet need for structured psychosocial support following MBS—including over the longer term(beyond 2 years post‐MBS) [15]. Thus, psychological vulnerabilities that were present prior to MBS (i.e., coping skill deficits, depression) coupled with various post‐operative stressors and limited options for support, may result in alcohol misuse to cope [11].

While a confluence of factors may collectively increase the risk for alcohol misuse post‐MBS [3, 4, 11], the more granular processes involved in alcohol use re‐initiation and escalation post‐MBS require further investigation. Prior work suggests an increase in the onset of AUD in the second year after MBS, while there is also evidence to suggest that the risk of developing AUD continues to increase up to 10 years post‐operatively [3, 4, 19]. Though some work has investigated factors associated with the re‐initiation of alcohol use post‐operatively [8], this work is sparse and even less is known about influences on the course of drinking over time following MBS. Exploring specific environmental (e.g., social situations) and cognitive‐behavioural (e.g., beliefs about alcohol use after MBS) drivers of post‐MBS alcohol use, from the first post‐MBS alcoholic drink to more frequent use, may illustrate modifiable risk and maintaining factors at vulnerable time points. Relatedly, there may be important differences in these factors among those with higher alcohol use or alcohol misuse relative to those with lower alcohol use. Thus, this qualitative study aims to (1) build on prior work to understand the course of post‐MBS alcohol use from reinitiation to continued episodic use (i.e., alcohol use on a given day), and escalation to alcohol misuse, (2) understand decision‐making processes associated with each phase, and (3) identify patterns or features that may be germane to higher post‐MBS alcohol use. Findings may identify important variables to investigate further at relevant time points in subsequent longitudinal and fine‐grained (e.g., daily or ecological momentary assessment) studies of post‐MBS alcohol use. Longer term, these research efforts are intended to support the development of specific, patient‐centred, and time‐sensitive AUD prevention and early intervention efforts.

## 2 Materials and Methods

### 2.1 Study Design, Setting, and Participants

This study was approved by the Henry Ford Health's Institutional Review Board (IRB #16312) and all participants provided informed consent. Patients who underwent MBS (i.e., sleeve gastrectomy or Roux‐en‐Y gastric bypass) between October 2018 and March 2023 at a single large healthcare system in the United States were randomly selected and invited to participate in the study, which involved completing a 1‐h semi‐structured interview. Patients were contacted by email, and those interested completed a brief eligibility screener via REDCap [20]. The screener inquired about demographic variables (i.e., age, gender, and race/ethnicity) to characterise the sample, and alcohol use over the past 3 months using an adapted version of the AUDIT‐C for the purposes of this study [21]. Specifically, instead of assessing past year alcohol use (item‐1), we asked about alcohol use over the past 3 months, given that some participants could be less than 12 months post‐operatively. Those who reported consuming alcohol at least 2–3 times per month over the previous 3 months were eligible. This inclusion criterion was chosen to ensure that participants had recent, consistent, and continued alcohol use post‐MBS. Of the 111 patients who completed the screener, 32 were eligible, and 20 consented. The enrolment goal was 20 participants to ensure an appropriate sample size to reach saturation [22].

### 2.2 Data Collection

An interview guide was collaboratively developed by experts in MBS, AUD and qualitative methods. The approximately 60‐min semi‐structured interview was conducted by a trained member of the study team. Themes related to initiation (e.g., ‘Why do you think people who have had MBS start drinking after surgery?’; ‘When did you first have alcohol after MBS?’), episodic use (e.g., ‘On any given day, what situations do you think lead someone to drink alcohol after MBS?’), escalation (e.g., ‘At what point had you progressed from 1 sip to a full drink, to multiple drinks?’; ‘When might someone develop a problem with alcohol?’), and contributors to problematic alcohol use (e.g., ‘Why do you think some people develop problems with alcohol after MBS?’) were explored. Both direct and indirect questions about participants' personal experiences and their perspectives on others' alcohol use post‐MBS were included across themes. We opted for this approach because alcohol use and misuse are sensitive and stigmatised topics in the general population, and this is likely heightened among individuals who have undergone MBS (as many are advised to limit or abstain from alcohol entirely) [3, 23]. Further, some questions—particularly those regarding alcohol misuse—may not have been applicable to all participants, or they may not have perceived them to be personally applicable. Therefore, the interview was intended to provide flexibility and the option to discuss these topics without disclosing their personal experience ifthey werenot comfortable or if the questions were not personally relevant. We anticipated that participants would still share their own experiences when appropriate, which was often the case (described in Results). These indirect questioning techniques have been empirically supported for similar qualitative research purposes (i.e., when questions may not be directly relevant for participants, or when their responses may be influenced by social desirability bias) [24]. These strategies can elicit findings that are comparable to direct questioning techniques, while potentially fostering greater comfort and supporting participants' autonomy andprivacy during the interview process [24, 25].

### 2.3 Data Analysis

Interviews were audio‐recorded and transcribed. Two raters independently read all 20 transcripts, and iteratively coded transcripts using Dedoose software [26]. Coding meetings occurred regularly between the two raters to discuss each transcript. Any discrepancies in coding were discussed until the raters agreed. Though a third person was available as a tie breaker, this was not needed. A primarily deductive approach was used based on pre‐existing goals of the research team and was informed by relevant literature [13, 14, 15, 16, 17, 18, 27, 28]. A formal codebook was developed by the clinically trained researchers to guide the coding. Themes across the major categories included (1) Timing and pacing of post‐MBS reinitiation and escalation of alcohol use, (2) Decision making processes related to post‐MBS alcohol use and (3) Participants' perspectives on the contributors to alcohol misuse or AUD post‐MBS. Participants were stratified by their average alcohol use. Higher alcohol use was categorised as 3–4 drinks per sitting and/or drinking at least 2–3 times per week. Lower alcohol use was considered 1–2 drinks/sitting with a frequency of once/week or less, but at least 2–3 times per month. This determination was informed by guidelines from the National Institute of Alcohol Abuse and Alcoholism for the general population, as there are no safe or appropriate levels of alcohol use that have been determined for those who have undergone MBS [29]. Findings for the first and second themes were stratified to identify potential group differences. The third theme was more general as participants were not necessarily commenting on their own experiences with alcohol misuse and therefore these results were not stratified.

## 3 Results

Participants were 95% female (*n* = 19) with a mean age of 44.1 years (SD = 10.7). Self‐identified racial groups included White (55%, *n* = 11), Black/African American (35%, *n* = 7), multiracial or other (10%, *n* = 2). Participants underwent sleeve gastrectomy (*n* = 14) or Roux‐en‐Y gastric bypass (*n* = 6) and were between 6 months and 5 years post‐operatively (25% were 4–5 years, 20% were 3–4 years, 30% were 2–3 years, 15% were 1–2 years, and 10% were 6–12 months post‐operatively). There were 11 participants (55%) who endorsed high alcohol use and 9 participants (45%) reported lower alcohol use. Across time points post‐operatively, all participants 4–5 years post‐operatively were in the high alcohol use category (*n* = 5), compared to 50% of those in their third year (*n* = 2), 16.7% of those in their second year (*n* = 1), 66.7% of those in their first year (*n* = 2) and 50% of those between 6 and 12 months post‐operatively (*n* = 1). In response to a combination of indirect and direct questioning techniques, participants mostly described their own experiences when applicable. However, responses often focused on others' post‐MBS alcohol use (whom they often knew personally) or consisted of speculations about others more broadly post‐MBS in response to questions related to alcohol misuse. These questions may not have been applicable to them; they may not have perceived them to be personally applicable (i.e., if they did not believe they misused alcohol), or they may not have been comfortable disclosing personally relevant experiences (Theme 1, section 3.1: escalation to alcohol misuse, and Theme 3, section 3.3: contributors to alcohol misuse). However, even responses focused on others—or their assumptions about others' experiences—were often informed by their unique post‐MBS perspectives and consideration of relevant contextual factors. Thus, these observations and speculations still yielded valuable insights.

### 3.1 Theme 1: Timing and Pacing of Reinitiation and Escalation of Post‐MBS Alcohol Use

Many (*n* = 10; 7 high alcohol use) indicated that they reinitiated alcohol between 2 and 7 months post‐operatively, while others (*n* = 8; 4 high alcohol use) reported drinking approximately 1 year after MBS, and only 1 reported reinitiation of drinking approximately 2 years following MBS (lower alcohol use). Nearly all participants indicated that when reinitiating alcohol use, they started slowly (*n* = 19; 12 high alcohol use). This often included starting with a few sips and gradually increasing the amount over time due to changes in the impact of alcohol after MBS (i.e., decreased tolerance/increased sensitivity or feeling intoxicated more quickly).

Yeah, totally very slow. I was just—like I said, it was probably maybe four ounces. And then it kind of grew, kind of grew, and then the glasses got bigger (laughs). … And I went from, you know, maybe just like the 4 ounces just that one evening to like now I can down a bottle of wine in an evening. (high alcohol use)

For some, increasing to more consistent or episodic use occurred within 3 weeks to 4 months after initial reinitiation (*n* = 8; 4 high alcohol use), and over a period of greater than 4 months for others (*n* = 6; 3 high alcohol use).

… I mean I think it was probably a matter of four‐six months where I was being really careful and then, just was like, ‘*Okay, everything else seems to be okay*’. And then gradually started drinking a little bit more throughout that time. (high alcohol use)

A few participants thought that escalation to alcohol misuse was likely to occur quickly (i.e., within a few months) (*n* = 3), while another was unsure and thought it was dependent on the individual (*n* = 1) (all high alcohol use).

I think you can develop a problem with alcohol pretty fast because your stomach is so much smaller and I think the more you drink, the quicker you can get that addiction. I think the addiction would be faster for someone who has the surgery because it takes a smaller amount to get drunk. (high alcohol use)

### 3.2 Theme 2: Decision Making Processes Related to Post‐Operative Alcohol Use

The role of pre‐operative intentions, beliefs and attitudes towards the risks or acceptability of post‐MBS alcohol use, and factors influencing continued episodic use were explored.

#### 3.2.1 Pre‐operative Intentions for Post‐MBS Alcohol Use

Most participants indicated that prior to surgery, they had no intentions of maintaining lifelong abstinence from alcohol (*n* = 15; 7 high alcohol use). They acknowledged that it seemed unrealistic never to drink again after MBS. Thus, old habits or personal norms for alcohol use were relevant to their decision to re‐engage in post‐MBS alcohol use.

I knew I couldn't drink initially after the surgery, so I would have to wait because of health things. But it was never my intention to be like, ‘*Oh, I'm never going to drink again*’. I knew I would still drink socially. So that's pretty much why I started back to drink. It was just kind of like, ‘*Well, I'm going on vacation. It's my husband's birthday. I want to have a drink with him*’. (lower alcohol use)

However, a few participants (*n* = 4; all high alcohol use) were unsure or thought intentions for post‐MBS alcohol use varied based on individual factors, and 1 individual indicated that they did plan to abstain from alcohol post‐MBS.

No, I think they (plan to) abstain. They think they're going to abstain. I know in my mind, I did. I was like, ‘*Oh, I don't care if I ever drink again*’. … That was my mindset. (high alcohol use)

#### 3.2.2 Beliefs and Attitudes About Post‐MBS Alcohol Use (Risks/Harm Versus Acceptability/Tolerability)

Beliefs and attitudes about the risks of post‐MBS alcohol use were influenced by interpretations of others' experiences (*n* = 15; 9 high alcohol use), and medical or online sources (*n* = 13; 9 high alcohol use). Many also felt that post‐MBS alcohol use was acceptable or that the risk of harm seemed low (*n* = 15; 12 high alcohol use), yet several also described feeling cautious about post‐MBS alcohol use (*n* = 12; 6 high alcohol use). Of note, responses that referenced others' experiences were described as they related to and informed their perceptions about the risks (or lack there of) of post‐MBS alcohol use.

My mom did not drink. My brother and sister‐in‐law and best friend all did. I don't know the timeframes of how long they waited because I know it was a minute. But then they would drink, not daily, but probably casually. And they never said that anything—like anything negative happened. (high alcohol use)

I think primarily right after surgery, I was really sort of cautious about it. I do have a family history of substance use and addiction disorders. So, I have always sort of been cautious. But (the surgery) made me even more cautious as far as knowing that it could affect me and impact me more now that my body was responding differently to substances. (lower alcohol use)

For some, initial fears or reservations about post‐MBS alcohol use appeared to be mitigated by recognising that their body could tolerate small amounts of alcohol with no major observable consequences—or believing that their alcohol use would not become problematic.

… So, I think after surgery I was scared to start drinking ‘cuz I didn't know how it would affect me. But then once I learned how it does affect me, then, you know, then I was okay with it. (high alcohol use)

So, I didn't want to turn into an alcoholic because I knew I had a food problem, so that's why I waited a whole year because I was scared. And then I decided to have one glass of wine. … I thought, ‘*I'm not drinking because I'm stressed, or anxious—I'm just drinking because I went out to dinner. I'm not drinking because I have a problem, so this is not going to turn into a problem*’. (high alcohol use)

And similarly: ‘I know for me—I always enjoyed it before. It was never a problem before. And I felt I could control it’. (high alcohol use)

Information about post‐MBS alcohol use was also obtained from online sources (i.e., Google or social media) (*n* = 11; 8 high alcohol use) and medical sources (i.e., from their dietician or a professionally led MBS educational group) (*n* = 2; 1 high alcohol use).

You know, there was a Facebook group. I don't even remember what it was called. … It was more of like a support group. And people were very candid about things they could do, things they couldn't do post‐operatively. Alcohol came up quite a bit. And food and stuff. But that was really my only (source of information) between my friends and that. (high alcohol use)

… I just listened to my doctor's recommendation because it scared me. I felt like something was going to rupture. Like I just went through blood, sweat and tears to have this surgery and I didn't want to mess it up. (lower alcohol use)

#### 3.2.3 Factors Influencing Continued Episodic Use

When asked about whether their episodic alcohol use was typically planned or unplanned, most indicated that it was often unplanned (*n* = 16; 8 high alcohol use), and others (*n* = 9; 6 high alcohol use) indicated that it was sometimes planned, or a combination of both. Anticipating alcohol to be a component of social events or outings (*n* = 3; 2 high alcohol use), accounting for other responsibilities (i.e., children, transportation) (*n* = 1; high alcohol use), experiencing stress earlier in the day (*n* = 1; high alcohol use) or routine (i.e., planned alcohol use on weekends) (*n* = 1; lower alcohol use) were relevant to planned use.

Earlier in the day. … But if I knew like, ‘*Okay, I am having a more stressful day*’, I'd say it's probably about like two hours beforehand I know, ‘*All right. I'm going to make an order when I get my groceries'* or letting my spouse know that he needs to pick something up. (high alcohol use)

I think it depends. For the most part, it's a preplanned thing of, ‘*Oh, I have lunch plans with So‐and‐So at the end of the week*’ and I'm already planning to go have margaritas and get lunch with my friend. But there are definitely sometimes where it's like a Friday evening and I'm like, ‘*You know what? I feel like having a drink tonight*’. And we have a drink and it's fine. So, it's a little bit of both, but more so that preplanning piece. (lower alcohol use)

Contributors to unplanned episodic use included social opportunities (*n* = 5; 3 high alcohol use), mood (*n* = 3)—whether positive (*n* = 2; lower alcohol use) or negative (*n* = 1; high alcohol use), and visual triggers or availability (e.g., seeing it on the counter or at the store) (*n* = 3; 2 high alcohol use).

So for me, I—that's a good question because I have been trying to figure it out so I can stop. I will see the bottle sitting on the countertop and just walk by and I'm like, ‘*Oh! It's sitting here. I'm going to have a drink*’. And so I pour myself a drink. So it's visual. (high alcohol use)

Like a few weeks ago—we were celebrating—I hadn't planned on drinking this day. We were celebrating a coworker's last day. ‘*Oh, we're going to go here after work*’. So, I went. … The plan was to go and eat, but once I got there, it was happy hour, so it was like, ‘*Oh, I'll try this*’. (lower alcohol use)

### 3.3 Theme 3: Contributors to the Development of Alcohol Misuse

Participants speculated about why some individuals might experience misuse or develop an AUD post‐MBS. These findings were not stratified as participants were not necessarily describing their personal experiences with alcohol misuse—however, they may have still referenced relevant post‐MBS contextual factors or aspects that they could relate to or felt comfortable disclosing in their responses. Participants postulated that alcohol misuse or AUD may develop due to having a propensity towards addiction or using alcohol to cope (*n* = 10).

So I do have one friend … we have children that are the same age and she had the surgery, and she is a very heavy drinker. And I do think that she drinks a lot because she's in a miserable marriage and, she's very down. And alcohol makes her feel better. She even says, ‘*I don't have to drink a lot, and it just makes me feel better*’. I have been there, you know … I do feel better having this glass of wine because I am upset about something. It puts me in a better place. At the risk of sounding cliché, it really does numb pain.

Biological changes (i.e., increased sensitivity to alcohol, possible vitamin deficiencies) (*n* = 6), were noted as a possible explanation for alcohol misuse. Old habits or a history of alcohol misuse without adequately accommodating for post‐MBS differences in alcohol sensitivity, was also noted as a potential contributing factor (*n* = 5). Lastly, some described a level of awareness and self‐monitoring of their drinking due to the potential for alcohol misuse, given individual risk factors (i.e., family history of addiction) or noticing certain habits (*n* = 5).

I definitely think because you get drunk fast and then you—I do notice that you get sober. … I know as for me in my experience, I did get intoxicated a bit faster … so that could cause—lead to someone wanting more because they're not maintaining the buzz they had …

I will say that I'm like a lot more conscious now of little habits I have picked up on, and I'll pay attention to it. Like one week I opened a bottle of wine, so I had the wine in the refrigerator, and I didn't want it to go flat so it was like once a day, I would drink a glass of wine. And I said, ‘*Wait a minute. This is getting to be too much*’. Like, ‘*I don't want to pick up this habit of like doing this every day and forming an addiction to it*’.

## 4 Discussion

Individuals within 5 years post‐MBS endorsing regular alcohol consumption offered valuable insights on the course of their post‐operative alcohol use and relevant factors at key phases (i.e., reinitiation, continued episodic use, and escalation). Most indicated that they began drinking alcohol within one year post‐MBS, started with small quantities, and increased gradually. Over half reported drinking at high levels, and all participants 4–5 years post‐operatively endorsed high alcohol use. While several themes did not differ widely between those drinking at higher versus lower levels, reinitiating alcohol less than 12 months post‐MBS, and the perception that post‐MBS alcohol use was not harmful, were disproportionately reported by those with high alcohol use. While risk factors for the development of alcohol misuse from participants' perspectives were largely consistent with prior work among this population (e.g., [3]), our findings add context to the rate at which these problems may develop, and provide unique insight into some participants' self‐monitoring processes. Further, participants' insights shed light on the role of cognitive‐behavioural factors underpinning reinitiation and continued alcohol use post‐MBS and may illustrate patterns associated with higher alcohol use. Ultimately, findings may inform subsequent quantitative and longitudinal research with larger samples, as well as AUD prevention and intervention efforts for individuals following MBS.

### 4.1 Timing and Pacing of Post‐MBS Alcohol Use

Half of our study participants reported having their first drink of alcohol between 2 and 7 months after MBS, and 70% of these individuals were currently drinking at high levels. Prior work often focuses on rates of alcohol use starting at least 1 year after MBS [2, 3], as patients may be advised to either abstain from alcohol within the first 6–12 months or altogether post‐MBS [3, 23, 30]. While the timing of initial alcohol use post‐MBS varies, our findings are consistent with work suggesting that a substantial proportion of individuals may initiate alcohol use less than 1 year after the surgery [3, 7]. Reinitiating alcohol use within the first year may partially explain why there is a rise in AUD diagnoses in the second year post‐MBS [3, 4]. However, participants indicated that problems with alcohol could develop at an accelerated pace post‐MBS due to biological changes, lack of awareness, and attempting to drink as they did previously—and therefore a small number may be vulnerable to developing AUD within the first year. Relatedly, although definitive conclusions cannot be drawn given the small sample, of the only two participants in our sample who were within 6–12 months post‐operatively, one was already drinking at high levels. Thus, future well‐powered quantitative research is needed to clarify the rate at which AUD develops after reinitiation ofalcohol use post‐operatively.

Although limited to a small sample, all participants who were 4–5 years post‐MBS at the time of the interview were drinking at high levels, compared to only a subset of those 3 years or less. This is consistent with work identifying that the risk of hazardous drinking and AUD continues to rise as time elapses from MBS rather than plateau [2, 3, 4]. While one may engage in infrequent or light alcohol use in the initial years following MBS and may not appear to be at risk for AUD, a large portion of those consuming alcohol post‐MBS may be vulnerable to engaging in risky levels of drinking over time. Further, while most described a slow and gradual reintroduction to alcohol post‐MBS in terms of amount and frequency, all participants were engaging in regular alcohol use, and more than half were drinking at high levels. Ultimately, although more research is needed with larger sample sizes, our findings integrated with prior work suggest a few features related to the timing and pacing of post‐MBS alcohol use, which may inform clinical practice. Primarily, engaging in alcohol use less than a year post‐MBS may be an early risk factor for high levels of drinking. Thus, while some MBS programs may recommend a slow and gradual reintroduction to alcohol post‐MBS, this precaution alone may not buffer the risk of developing alcohol misuse. Further, escalation to alcohol misuse was thought to develop at an accelerated pace–potentially within months. Lastly, the risk for developing alcohol misuse continues to rise over time. Therefore, individuals may benefit from earlier screening than previously anticipated (i.e., within the first 6–7 months post‐operatively) and frequent, ongoing monitoring of alcohol use up to a minimum of 5 years after MBS [2, 3, 4].

### 4.2 Factors Influencing Alcohol Use at Each Phase

Factors that were relevant to the decision to reinitiate alcohol use after MBS included pre‐operative intentions and beliefs about the risks or acceptability of post‐MBS alcohol use. Consistent with prior research, most participants did not believe that lifelong abstinence from alcohol was realistic, due to the role of alcohol in their own lives and societally [8, 23]. Thus, most participants intended to reinitiate alcohol use following MBS, often citing its relevance to social situations, events, one's mood, or previous habits. However, the intention to reinitiate alcohol use post‐MBS did not correspond with higher levels of drinking. Conversely, the one individual that did plan to abstain from alcohol, and the few that were unsure, were all drinking at high levels. Thus, even those who are more receptive to abstinence may be at risk for heavy alcohol use following MBS.

Beliefs and attitudes about post‐MBS alcohol use were shaped by learning about others' post‐MBS experiences (i.e., those they knew or through social media), evaluating their own early reactions to alcohol post‐MBS, and information obtained from medical sources or their providers. Several reported being hesitant, fearful or cautious about post‐MBS alcohol use—particularly in the early stages of reinitiation. Some described a discrepancy between their expectations of the medical consequences (e.g., believing that they would be physically unable to drink, or that something would ‘rupture’), and the reality of their experience or others' experience (e.g., that they were still physically able to drink and tolerate small amounts of alcohol). Relatedly, some described a shift from initial fears about the consequences, to appraising alcohol use as low risk or acceptable. The perception that there was minimal harm associated with post‐MBS alcohol use was primarily endorsed by those drinking at higher levels (i.e., comprising 80% of those expressing this belief or attitude). Thus, this may be one feature associated with higher alcohol use post‐MBS. Taken together, findings on the role of pre‐operative intentions and beliefs/attitudes about post‐MBS alcohol use suggest that it may be important to: (1) evaluate patients' pre‐operative intentions for post‐MBS alcohol use, (2) consider the sources of information influencing patients' beliefs and intentions for post‐MBS alcohol use, and (3) ensure patients have an accurate understanding of the wide‐ranging risks associated with post‐MBS alcohol use—even in the absence of immediately observable consequences. Assessing this among MBS patients may provide an opportunity for, or inform, individualised AUD prevention or early interventions pre‐ or post‐operatively. Fortunately, the belief that post‐MBS alcohol use is low‐risk may be relatively malleable with the utilisation of relevant clinical strategies (i.e., cognitive‐behaviour therapy, motivational interviewing) and providing accurate information about the risks [31, 32, 33].

Consistent with overarching reasons for reinitiation, continued episodic alcohol use post‐MBS was influenced by social circumstances and mood. Visual triggers or cues—such as having alcohol in the home or seeing it in the grocery store, were also relevant and unique to episodic use—particularly unplanned use. Although both planned and unplanned episodic use were common and did not differ widely between those drinking at lower versus higher levels, there may be subtle differences in the nature and reasons for episodic use among those with alcohol misuse or AUD post‐MBS worth investigating in future research. Participants speculated about potential biopsychosocial contributors to the development of alcohol misuse post‐MBS—which were largely consistent with risk factors that have been identified in quantitative research [3, 13]. In addition, some participants described a degree of awareness and self‐monitoring of potential misuse due to pre‐existing risk factors (i.e., family history of addiction) or in response to observing new habits. This adds to prior work by illustrating a cognitive‐behavioural process that may potentially reduce the risk of AUD. For example, building on this skill of self‐monitoring (i.e., monitoring their frequency of alcohol use, identifying triggers or antecedents for use, etc.), promoting awareness of the warning signs of alcohol misuse, and providing resources should they notice these signs both pre‐operatively and post‐operatively, may be crucial to reducing AUD risk while supporting patients' autonomy [31]. Technology‐based approaches that build on these principles designed to reduce post‐MBS alcohol use may be particularly well‐suited to address nuanced, modifiable risk factors in this population, as a standalone or adjunctive intervention option within MBS programs [32, 34].

### 4.3 Strengths and Limitations

A strength of this study was leveraging qualitative methods to explore the process of reinitiation and escalation of alcohol use among those 6 months to 5 years post‐MBS who endorsed regular drinking. Findings meaningfully build on prior work related to the incidence and risk factors for post‐MBS AUD by exploring more granular aspects associated with the course and progression of alcohol use from patients' perspective. Stratification of results by level of alcohol use enabled us to explore how responses differed between groups, and identify potential patterns associated with higher use. However, this was done with a small sample, and we were unable to make statistical comparisons. Further, given our eligibility criteria, findings may not be generalisable to the broader population of those who undergo MBS, and responses would likely differ among those who consume alcohol infrequently. While more women tend to undergo MBS than men, given that our sample was predominantly women (i.e., only 1 male) findings may also differ significantly among men post‐MBS given gender differences in AUD and patterns of alcohol use [35]. Further, interpretations may be limited by varying levels of insight from participants regarding their alcohol use, and a tendency to respond in a socially desirable manner due to associated stigma. Our use of indirect questioning techniques was intended to reduce this potential for stigma and social desirability bias by offering flexibility for participant responding. However, given that participants at times commented on others' behaviour rather than their own, particularly for questions related to alcohol misuse, there are limitations to what can be inferred from these responses and therefore these findings should be interpreted with caution. Lastly, while there are some studies that suggest that Roux‐en‐Y gastric bypass may have more pronounced impacts on responses to alcohol or stronger associations with AUD risk (e.g., [36]), there is also evidence to suggest that the effects and increased risk of alcohol misuse are comparable for sleeve gastrectomy and gastric bypass, particularly when using sensitive forms of measurement [6, 37]. However, given the potential for subtle or nuanced differences in post‐MBS alcohol use and misuse by procedure type, this should be investigated in future work with larger samples sufficiently powered to identify these potential differences.

### 4.4 Conclusions and Implications for Continued Research

**FIGURE 1:**
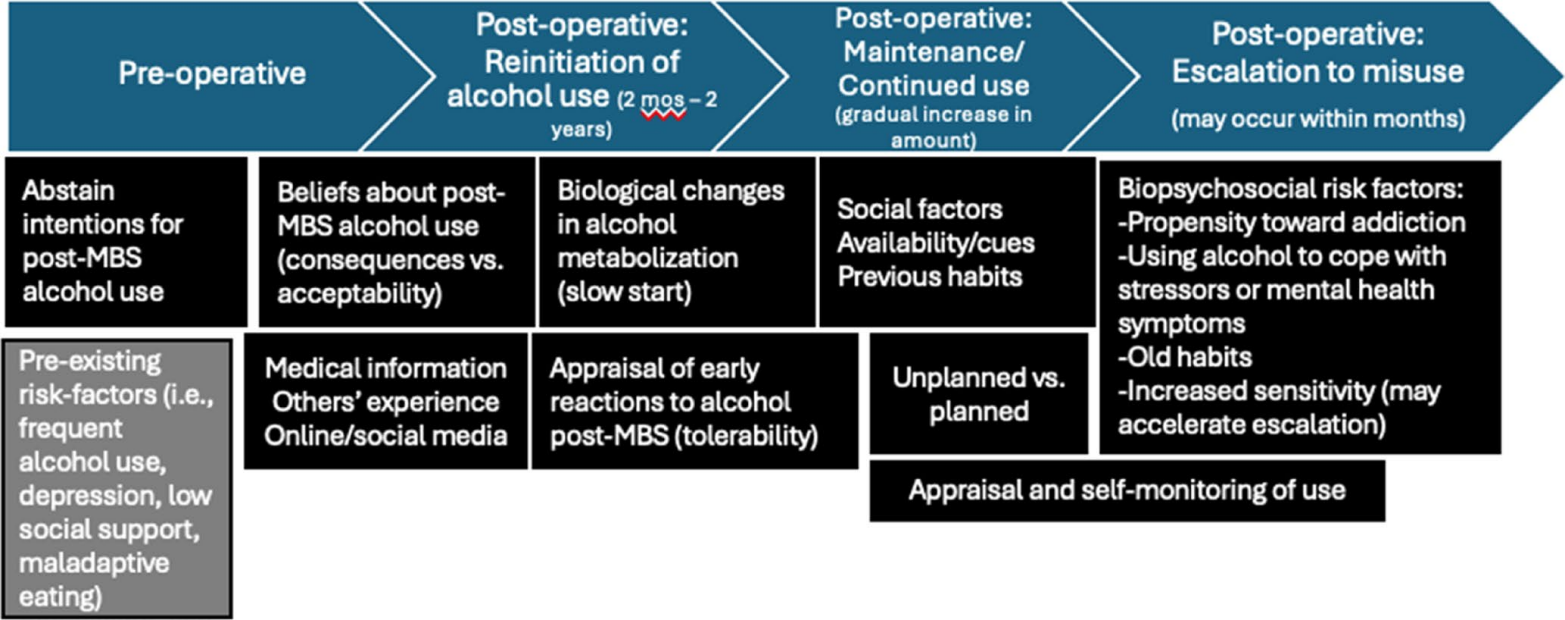
Preliminary conceptual model of factors relevant to the course of post‐MBS alcohol use.

Individuals 6 months to 5 years post‐MBS provided key insights into factors shaping their post‐MBS alcohol use, considerations for the timing and progression of these phases, and patterns that may be pertinent to higher alcohol use. While more research is warranted, findings suggest a few critical considerations for post‐MBS care. First, reinitiating alcohol use less than 12 months post‐operatively may indicate a risk for higher alcohol use, and starting slowly does not necessarily mitigate this risk. Relatedly, alcohol use may continue to increase over time rather than plateau, and escalation to misuse may occur quickly. Finally, there are several potential intervention targets that can be addressed pre‐ and post‐operatively (i.e., beliefs about post‐MBS alcohol use, behavioural self‐monitoring of use, and using alcohol to cope with stressors). These findings, integrated with prior work, may inform hypotheses about relevant mediating or moderating variables to investigate in subsequent longitudinal research on the development of alcohol misuse and AUD post‐MBS (Figure 1).

## Author Contributions

E.N.H.: conceptualisation, original draft preparation, writing, review and editing. A.M.V.: data collection, original draft preparation, writing, review and editing. J.M.B.: methodology, data collection and analysis, review and editing. L.R.M.‐M.: conceptualisation, methodology, data collection and analysis, writing, review and editing. All authors participated in the writing, review, and editing of the paper, and approved the final manuscript.

## Funding

Funding for the research was obtained from the National Institute of Health (R21AA029423).

## Conflicts of Interest

The authors declare no conflicts of interest.

## Data Availability

The data are not publicly available due to privacy or ethical restrictions. Because of the qualitative nature of this work and to protect participant privacy, transcripts will not be shared. The interview guide and the codebook can be shared upon request.
